# Pharmacogenomic heterogeneity of *N-acetyltransferase 2*: a comprehensive analysis of real world data in Indian tuberculosis patients and from literature and database review

**DOI:** 10.1080/07853890.2025.2478316

**Published:** 2025-03-26

**Authors:** Levin Thomas, Chaithra, Yashi Batra, Mitali Mathur, Shrivathsa Kulavalli, Chidananda Sanju SV, Naveen Dutt, Pankaj Bhardwaj, Muralidhar Varma, Kavitha Saravu, Mithu Banerjee, Mahadev Rao

**Affiliations:** a Department of Pharmacy Practice, Manipal College of Pharmaceutical Sciences, Manipal Academy of Higher Education (MAHE), Manipal, India; b Department of Biochemistry, All India Institute of Medical Sciences, Jodhpur, India; c Department of Microbiology and Molecular Genetics, Oklahoma State University, Stillwater, OK, USA; d District Tuberculosis Control Office, Udupi, India; e Department of Pulmonary Medicine, All India Institute of Medical Sciences, Jodhpur, India; f Department of Community Medicine and Family Medicine, All India Institute of Medical Sciences, Jodhpur, India; g Department of Infectious Diseases, Kasturba Medical College, Manipal, Manipal Academy of Higher Education, Manipal, India

**Keywords:** Isoniazid, *N*-acetyltransferase 2, pharmacogenomics, single nucleotide polymorphism, AT-DILI

## Abstract

**Background:**

Isoniazid is primarily metabolized by the arylamine *N*-acetyltransferase 2 (NAT2) enzyme. Single nucleotide polymorphisms (SNPs) in the *NAT2* gene could classify an individual into three distinct phenotypes: rapid, intermediate and slow acetylators. *NAT2* SNPs and the slow acetylator phenotype have been implicated as risk factors for the development of antitubercular drug-induced liver injury (AT-DILI) in several tuberculosis (TB) populations.

**Patients and methods:**

We conducted a prospective observational study to characterize and compare the *NAT2* SNPs, genotypes and phenotypes among patients with TB and AT-DILI from the Southern and Western regions of India. The *NAT2* pharmacogenomic profile of patients from these regions was compared with the reports from several geographically diverse TB populations and participants of different genetic ancestries extracted from literature reviews and the ‘All of Us’ Research Program database, respectively.

**Results:**

The TB patients of Southern and Western regions of India and several other geographically closer regions exhibited near similar *NAT2* MAF characteristics. However significant heterogeneity in *NAT2* SNPs was observed within and between countries among AT-DILI populations and the participants of different genetic ancestry from the ‘All of Us’ Research Program database. The MAF of the *NAT2* SNPs rs1041983, rs1801280, rs1799929, rs1799930 and rs1208 of the TB patients from Southern and Western Indian Sites were in near range to that of the South Asian genetic ancestry of ‘All of Us’ Research Program database. About one-third of the total TB patients from the Southern and Western regions of India were *NAT2* slow acetylators, among whom a relatively higher proportion experienced AT-DILI.

**Conclusion:**

Further studies exploring the risk of *NAT2* SNPs in different AT-DILI patients with larger sample sizes and a population-specific approach are required to establish a policy for *NAT2* genotyping as a pre-emptive biomarker for AT-DILI monitoring for personalized isoniazid therapy in clinics.

## 1. Background

*N*-acetyltransferase 2 (NAT2) enzyme is involved in the biotransformation (phase II) of several xenobiotics such as aromatic amine, heterocyclic amine, or hydrazine compounds as well as several carcinogens present in the diet, cigarette smoke and in the environment, by catalysing the transfer of an acetyl group (−COCH_3_) from acetyl-coenzyme A to the target molecules [1–6]. Isoniazid, a first-line antitubercular drug (also known as isonicotinylhydrazine or INH), is predominantly metabolized (50%- 90%) in the liver and intestines by the NAT2 enzyme [7,8]. Suboptimal isoniazid plasma concentrations have been reported in several tuberculosis (TB) populations, warranting the need for higher isoniazid doses to improve the treatment outcomes [9–11]. Whereas TB patients experiencing antitubercular therapy (ATT) induced adverse drug reactions (ADRs) had higher isoniazid plasma concentrations [12]. The variability in isoniazid pharmacokinetics in both TB patients and healthy volunteers has been majorly attributed to the *NAT2* single nucleotide polymorphisms (SNPs)/genotype [13,14]. Hence, assessing the *NAT2* SNPs and genotype frequency is a potential host-based omics approach for addressing the inter- and intra-individual isoniazid pharmacokinetic variabilities, isoniazid dose optimization and preventing/reducing the risk of isoniazid-induced ADRs in TB patients for precision therapy of isoniazid [15]. TB patients were also reported to have relatively higher NAT2 expression levels in natural killer (NK) cells and monocytes as compared to healthy volunteers, suggesting its potential role as an early biomarker in the innate immune response to *Mycobacterium tuberculosis* [16–18].

Pharmacogenomics aids in developing strategies for individualizing patient treatment through knowledge of human genome variations and their influence on drug responses. SNPs represent the most frequent genome variations [19,20]. Several SNPs have been identified in the *NAT2* gene and the most studied *NAT2* polymorphisms are rs1801279 (191 G > A), rs1041983 (282 C > T), rs1801280 (341 T > C), rs1799929 (481 C > T), rs1799930 (590 G > A), rs1208 (803 A > G) and rs1799931 (857 G > A). However, among these, the *NAT2* rs1801279 was reported to be absent in several Asian and Indian populations [21–23]. These SNPs in the *NAT2* gene may change the structural and functional effects of the NAT2 enzymes by causing substrate affinity/conformational changes, reducing catalytic activity and protein stability and enhancing protein degradation [24–28]. The *NAT2* phenotypes classified as rapid, intermediate and slow acetylators are derived from *NAT2* haplotypes/SNP combinations [29]. These *NAT2* acetylator phenotypes account for the trimodal isoniazid elimination pattern, with *NAT2* rapid acetylators having a twofold or threefold increase in isoniazid clearance, compared to *NAT2* slow acetylators [30]. Antitubercular drug-induced liver injury (AT-DILI) is a common ADR associated with antitubercular drugs such as isoniazid in TB patients. A meta-analysis reported the pooled incidence of AT-DILI of 11.50% (95%CI: 10.10%–12.97%) from 1990 to 2022 and showed an upward trend over time (*p* < .001) from 1999 to 2020 [31]. Assessment of risk factors associated with AT-DILI is imperative for reducing hospitalization, morbidities and mortality during the ATT in TB patients [32,33]. *NAT2* SNPs and slow acetylator phenotype have been associated with a higher risk of AT-DILI [34]. *NAT2* genotype-guided isoniazid dosing may result in lower incidences of AT-DILI or early treatment failure compared to traditional dosing regimens [35].

Significant *NAT2* genetic diversity exists among populations across diverse geographical regions of a country, ethnic backgrounds and populations of different countries, including South Asian populations [21,36,37]. India has a genetically heterogeneous population, encompassing over 4500 anthropologically distinct populations of various caste, tribe and religious groups that exhibit diverse cultural, social and biological behaviours, making it a valuable resource for exploring clinically actionable pharmacogenomic variants [38–40]. Significant differences in the allelic frequencies of several clinically actionable pharmacogenomic variants have been reported between the populations of geographically distinct regions of India and between the Indian and global populations [40–42]. Studies focusing on *NAT2* pharmacogenomic heterogeneity of geographically diverse TB populations of a high TB burden country like India are sparse, leaving gaps in understanding region-specific allelic variant frequency and variations and their potential implications such as in the occurrence of AT-DILI. We hypothesize significant *NAT2* SNP heterogeneity among TB and AT-DILI populations within diverse geographical regions of India and between Indian and other global TB populations. Further, we hypothesize that *NAT2* slow acetylators exhibit a higher risk of developing AT-DILI, irrespective of geographical diversity within India.

Hence, we aimed to investigate the *NAT2* SNP frequency in the TB populations of two geographically diverse regions (Southern and Western) of India. Such a multicentric geographically diverse exploration of the *NAT2* SNP is imperative for deriving a strong conclusive statement for the potential use of *NAT2* genotyping as a preemptive biomarker for AT-DILI monitoring for national-level policy implementation, consideration of geographic-specific *NAT2* SNPs for AT-DILI monitoring, as well as futuristic exploration of using *NAT2* genotype as a potential covariate for isoniazid dose optimization in TB patients. Further, we aimed to compare and characterize the *NAT2* pharmacogenomic diversity from our studied population with several populations of different countries through a literature review of the studies reporting on the *NAT2* SNP frequency in different TB and AT-DILI populations as well as from the participants of the ‘All of Us’ Research Program database from the Public Tier dataset. The ‘All of Us’ Research Program is a longitudinal cohort study with a clinical-grade genomic data release of about ∼245,000 individuals of diverse groups [43]. The ‘All of Us’ Research Hub has three data access tiers: a public tier dataset, a registered tier curated dataset and a controlled tier dataset. The public tier dataset contains only aggregate data with identifiers removed and is available to the public through Data Snapshots (https://www.researchallofus.org/data-tools/data-snapshots/) and the public Data Browser (https://databrowser.researchallofus.org/) [43].

## 2. Patients and methods

### 2.1. Pharmacogenomic profiling of *NAT2* in TB patients from Southern and Western regions of India

#### 2.1.1. Study population

A prospective observational multicentric study was conducted, including patients diagnosed with TB and initiated on isoniazid-containing ATT from December 2021 to March 2024. TB patients visiting Kasturba Medical College, Manipal (Southern Indian Site) and All India Institute of Medical Sciences (AIIMS), Jodhpur (Western Indian Site) were included using a convenient sampling method. The study adhered to the principles of the Declaration of Helsinki. The study received institutional ethics committee (IEC) approval from each Study Site (Kasturba Hospital and Kasturba Medical College IEC No. 243/2019 and AIIMS Jodhpur IEC No. 2019/3522). Written informed consent was obtained for all patients. The demographic and clinical data were extracted from medical records and patient interviews. Later in this study, TB patients who developed AT-DILI and met either of the DILI criteria laid by the American Thoracic Society or NIH Drug-Induced Liver Injury Network (DILIN) were also identified [44–46]. The serum samples were collected from these TB patients for *NAT2* genotyping and were stored at −80 °C until deoxyribonucleic acid (DNA) extraction.

#### 2.1.2. NAT2 genotyping

The QIAamp DNA blood mini kit (Qiagen, Hilden, Germany) was used to extract genomic DNA from the serum sample of the patients with TB according to the kit instruction manual. The extracted genomic DNA was quantitated on a NanoDrop 2000 spectrophotometer (ThermoFisher Scientific, U.S.A.). The genomic DNA samples were stored at −80 °C until analysis. Genotyping of the six SNPs in the *NAT2* exon 2 coding region, rs1041983, rs1801280, rs1799929, rs1799930, rs1208 and rs1799931 was performed using the TaqMan^®^ 5′-nuclease assay chemistry on QuantStudio^™^ 5 Real-Time PCR System (Applied Biosystems, U.S.A.). The predesigned TaqMan^™^ assay IDs of the six *NAT2* SNPs used for polymerase chain reaction (PCR) were C___8684085_20 (rs1041983), C___1204093_20 (rs1801280), C___1204092_20 (rs1799929), C___1204091_10 (rs1799930), C____572769_20 (rs1208) and C____572770_50 (rs1799931). The TaqMan probes, labelled with either VIC or FAM as a 5′ reporter dye and equipped with a nonfluorescent 3′ quencher with MGB, specifically target one of the two possible bases at each *NAT2* SNP position. The thermal conditions of the experiment consisted of a pre-reading stage at 60 °C for 30 s, a hold stage at 95 °C for 10 min; and a polymerase chain reaction (PCR) stage comprising of alternating 45 cycles of step 1 at 95 °C for 15 s and step 2 at 60 °C for 1 min and post-reading stage at 60 °C for 30 s. The PCR reaction mixture consisting of 20 ng of genomic DNA was used for each assay/per well on MicroAmp^™^ Optical 96-well reaction plate (Applied Biosystems, U.S.A.). In each experiment, control samples with no DNA template (negative control) were run to ensure that there was no amplification of any contaminating DNA. The genotype of each sample was determined by measuring the allele-specific fluorescence using QuantStudio^™^ Design and Analysis Software v1.5.2 (Applied Biosystems, U.S.A.) for allelic discrimination. The corresponding *NAT2* haplotypes and phenotypes were classified according to the consensus nomenclature of the human *NAT2* alleles haplotypes database. Assessment of the six SNPs in the *NAT2* coding region SNPs described above allows for identifying the reported human *NAT2* alleles. TB patients possessing two of the *NAT2* alleles associated with rapid acetylation activity (NAT2*4, NAT2*11, NAT2*12 and NAT2*13 families) are classified as *NAT2* rapid acetylators. TB patients with two alleles associated with slow acetylation activity (NAT2*5, NAT2*6, NAT2*7 and NAT2*14) are classified as *NAT2* slow acetylators, and TB patients with one rapid allele and one slow allele were classified as *NAT2* intermediate acetylators, as described in the consensus nomenclature [16,47,48]. The minor allelic frequency (MAF) was determined for each *NAT2* SNP by the formula, MAF= (2⋅n_BB_+n_AB_)/2N, where n_BB_, n_AB_ and N are the number of individuals homozygous for the minor allele, number of individuals heterozygous (counted as contributing half the value of the allele) and total number of individuals, respectively.

### 2.2. Review of literature

We conducted a comprehensive literature search to identify the MAF of the six *NAT2* SNPs rs1041983, rs1801280, rs1799929, rs1799930, rs1208 and rs1799931 among different TB populations in PubMed and Google Scholar. We also extracted data from the literature describing the comparative MAF of the six *NAT2* SNP between TB patients who developed AT-DILI (cases) and those who did not develop AT-DILI (controls) of different TB populations.

### 2.3. ‘All of Us’ Research Program database

The MAF of the six *NAT2* SNPs analysed from the TB patients of the current study were compared with those of the participants (n=∼245,400) of the ‘All of Us’ Research Program from the data browser of the single nucleotide variant (SNV)/Indel Variants (public tier) [49]. The MAF for the six *NAT2* SNPs [variant IDs 8-18400285-C-T (rs1041983), 8-18400344-T-C (rs1801280), 8-18400484-C-T (rs1799929), 8-18400593-G-A (rs1799930), 8-18400806-G-A (rs1208) and 8-18400860-G-A (rs1799931)] of the participants categorized into seven categories of genetic ancestry populations of African, East Asian, European, Latin American, Middle Eastern, South Asian and Remaining (individuals did not neatly fit the patterns of any of the genetic ancestry groups) were extracted. The MAF of the six *NAT2* SNPs of the combined Southern and Western Indian TB patients of the original research investigations were compared to the MAF observed among the different genetic ancestries in the ‘All of Us’ Research Program.

### 2.4. Statistical analysis

**Figure 1.**
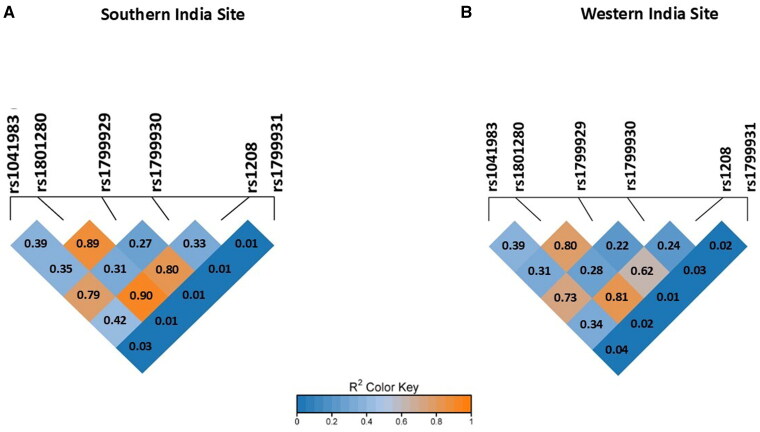
LD plot of TB patients from each study site. *Abbreviations:* LD: linkage disequilibrium. *Note*: The values inside the diamond-shaped boxes represent the r^2^ value between each pair of *NAT2* SNP.

**Figure 2.**
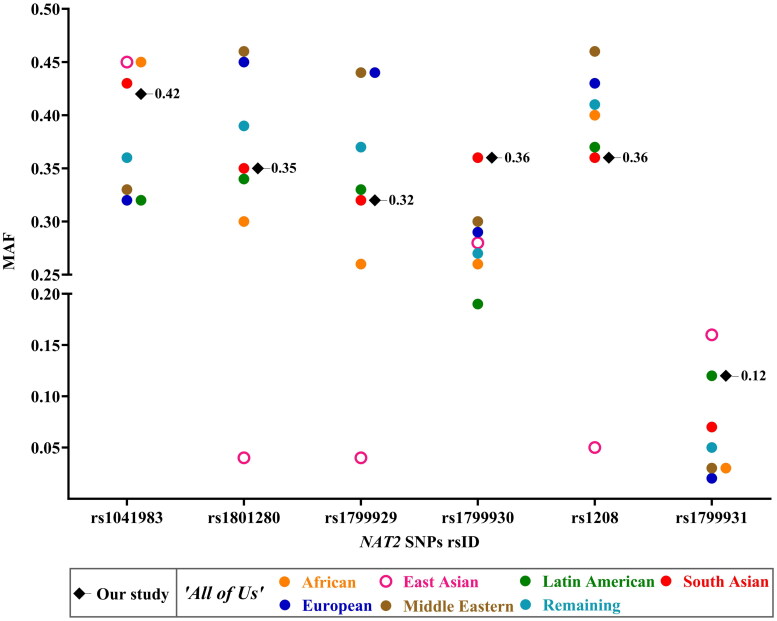
MAF of different *NAT2* SNPs across different participants of the ‘All of Us’ Research Program and current study (Southern and Western Indian Sites, *n* = 452). *Abbreviations:* MAF: minor allele frequency; *NAT2*: *N*-acetyltransferase 2; rsID: reference SNP cluster ID; SNP: single nucleotide polymorphism.

Descriptive statistics were used to summarize the demographic features of the TB patients. Frequency analysis summarized the *NAT2* SNPs, genotype and phenotype characteristics. The differences between the genotype frequencies of the TB patients with AT-DILI (TB + AT-DILI) and without AT-DILI (TB - AT-DILI) from the Southern and Western Indian Sites were determined using the Chi-square test or Fisher’s exact test (Table 1). Hardy-Weinberg equilibrium (HWE) states that genetic variation within a large, randomly mating population remains constant across generations in the absence of any disruptive evolutionary influences. HWE was used to evaluate the expected and observed distributions of genotypes in the TB population [50]. According to the HWE, the frequency of the three genotypes is determined by the binomial relationship (p + q)^2^ = 1, where p and q represent the frequency of two alternative alleles. The chi-square test was used to compare the observed genotype frequencies to those expected under HWE, with the test statistic calculated as χ2= $\sum \frac{{\left(0‐E\right)}^{2}}{E}$, where O and E represent the observed and expected genotype counts, respectively. SNPs with a Chi-square value of less than 3.84 at 1 degree of freedom (corresponding to a *p*-value > .05) were considered to be in equilibrium. The pairwise LD for the non-random association of the *NAT2* alleles at different loci was measured using the correlation coefficient (r^2^) using SNPstats, whose values range from 0 to 1, with 0 implying that the alleles are in complete linkage equilibrium and 1 implying that alleles are in completed LD [51,52]. The LD plot of the *NAT2* SNPs was generated using SRplot [53]. In the provided LD plots for 6 *NAT2* SNPs (Figure 1), each diamond-shaped box represents the pairwise LD value (measured as r^2^) between two SNPs. The colour of each box indicates the strength of LD between the two SNPs, as shown by the r^2^ colour key, with the dark blue (low r^2^) indicating weak or no LD and orange to red (high r^2^) indicating strong LD, suggesting the SNPs are likely inherited together. The plots generated for the MAF of the six *NAT2* SNPs among the different TB populations (Supplementary Figure 1), MAF of the six *NAT2* SNPs between TB patients who developed AT-DILI (cases) and did not develop AT-DILI (controls) (Supplemental Figure 2) and comparison of the MAF among the different populations from the ‘All of Us’ Research Program database with that of the TB patients of the Southern and Western Indian Sites (Figure 2) was generated using the GraphPad Prism Version 10.1.2 (GraphPad Software, USA).

**Table 1.** Frequency of *NAT2* SNPs in patients with TB and who developed AT-DILI from each study site.

*NAT2* SNP rsID (nucleotide position)	Genotype	Southern Indian Site							Western Indian Site						
		Total (N)	TB + AT-DILI (N)	TB-AT-DILI (N)	*p* Value	MAF (total)	MAF (DILI)	HWE (total)	Total (N)	TB + AT-DILI (N)	TB-AT-DILI (N)	*p* Value	MAF (total)	MAF (DILI)	HWE (total)
rs1041983 (282C > T)	CC	84	10	74	.327	0.43	0.50	0.51	69	0	69	.002*	0.41	0.68	0.24
	CT	117	22	95					101	9	92				
	TT	49	10	39					32	5	27				
rs1801280 (341T > C)	TT	111	14	97	.194	0.34	0.39	0.91	83	7	76	.398	0.36	0.25	0
	TC	106	23	83					93	7	86				
	CC	33	5	28					26	0	26				
rs1799929 (481C > T)	CC	116	15	101	.265	0.33	0.38	0.79	97	8	89	.610	0.31	0.21	0.04
	CT	104	22	82					85	6	79				
	TT	30	5	25					20	0	20				
rs1799930 (590G > A)	GG	101	15	86	.679	0.37	0.42	0.67	90	3	87	.168	0.33	0.46	0.01
	GA	111	19	92					89	9	80				
	AA	38	8	30					23	2	21				
rs1208 (803A > G)	AA	105	14	91	.277	0.36	0.39	0.78	80	7	73	.364	0.36	0.25	0.28
	AG	109	23	86					97	7	90				
	GG	36	5	31					25	0	25				
rs1799931 (857G > A)	GG	222	35	187	.279	0.11	0.17	250	174	9	165	.009*	0.14	0.32	193.58
	GA	0	0	0					1	1	0				
	AA	28	7	21					27	4	23				

*Abbreviations:* AT-DILI: antitubercular drug induced liver injury; HWE: Hardy Weinberg equilibrium (χ^2^); MAF: minor allele frequency; *NAT2*: *N*-acetyltransferase 2; rsID: reference SNP cluster ID; SNP: single nucleotide polymorphism. * refers to statistical significance (*p*< 0.05)

## 3. Results

### 3.1. Pharmacogenomic profiling of *NAT2* of TB patients from Southern and Western regions of India

The median [Q1, Q3] age in years of the TB patients from the Southern Indian Site (*n* = 250) and Western Indian Site (*n* = 202) was 47 [34.7, 57] and 33.50 [24.7, 50] years, respectively. There were 161 (64.4%) male and 89 (35.6%) female TB patients from the Southern Indian Site and 124 (61.4%) male and 78 (38.6%) female TB patients from the Western Indian Site. The median [Q1, Q3] BMI (kg/m^2^) was 19.7 [16.4, 22.4] (*n* = 216) and 19.5 [16.6, 22.5] (*n* = 193) of the Southern Indian and Western Indian Site respectively. There were 164 pulmonary TB (PTB), 61 extrapulmonary TB (EPTB), 6 PTB+EPTB, 11 disseminated TB and 8 miliary TB cases from the Southern Indian Site, whereas 94 PTB, 106 EPTB, 1 disseminated TB and 1 miliary TB cases from the Western Indian Site. The MAF of the six *NAT2* SNPs rs1041983, rs1801280, rs1799929, rs1799930, rs1208 and rs1799931 of the combined TB patients of Southern and Western India (*n* = 452) was 0.42, 0.35, 0.32, 0.36, 0.36 and 0.12, respectively. The MAF of these *NAT2* SNPs classified into respective Study Sites is shown in Table 1. Higher MAF was observed for all the six *NAT2* SNPs among TB patients who experienced AT-DILI (*n* = 42) relative to all the TB patients of the Southern Indian Site, whereas higher MAF was observed for rs1041983, rs1799930 and rs1799931 among the AT-DILI group (*n* = 14) compared to the all-TB patient’s group of the Western Indian Site as shown in Table 1. A statistically significant difference between the genotypes of the TB patients with and without AT-DILI groups was observed in the Western Indian Site for the *NAT2* SNPs rs1041983 and rs1799931. All the *NAT2* SNPs were in HWE except for rs1799931 in both centres.

The frequency of *NAT2* rapid, intermediate and slow acetylators was 5.6%, 62.4% and 32% in Southern Indian Site and 6.4%, 59.4% and 34.2% in Western Indian Site, respectively. *NAT2* slow acetylators had a relatively higher proportion of TB patients who developed AT-DILI in both Southern and Western Indian Sites as shown in Table 2.

**Table 2.** Frequency of *NAT2* phenotype and genotype among patients with TB and who developed AT-DILI from each study site.

		Southern Indian site					Western Indian site				
Sl. no	Genotypes	Total		AT-DILI		AT-DILI%: total%	Total		AT-DILI		AT-DILI%: total%
		N	%	N	%	Ratio	N	%	N	%	Ratio
*NAT2* rapid acetylators											
1	NAT2*4/*4	10	4	0	0	–	11	5.4	0	0	–
2	NAT2*4/*12A	4	1.6	0	0	–	2	1.0	0	0	–
Total		14	5.6	0	0	–	13	6.4	0	0	–
*NAT2* intermediate acetylators											
3	NAT2*4/*5A	0	0	0	0	–	2	1.0	0	0	–
4	NAT2*4/*5B	67	26.8	9	21.4	0.8	48	23.8	0	0	–
5	NAT2*4/*5C	3	1.2	1	2.4	2.0	6	3.0	0	0	–
6	NAT2*4/*6A	76	30.4	12	28.6	0.9	49	24.3	3	21.4	0.9
7	NAT2*4/*7B	6	2.4	0	0	–	10	5.0	2	14.2	2.8
8	NAT2*6A/*11A	1	0.4	0	0	–	0	0	0	0	
9	NAT2*6A/*12A	3	1.2	0	0	–	5	2.5	0	0	
Total		156	62.4	22	52.4	0.8	120	59.4	5	35.7	0.6
*NAT2* slow acetylators											
10	NAT2*5A/*6A	0	0	0	0	–	2	0.9	0	0	–
11	NAT2*5A/*7B	1	0.4	0	0	–	0	0	0	0	–
12	NAT2*5B/*6A	56	22.4	13	31.0	1.4	42	20.7	5	35.7	1.7
13	NAT2*5B/*7B	9	3.6	5	11.9	3.3	10	4.9	1	7.1	1.4
14	NAT2*5C/*6A	2	0.8	0	0	–	8	3.9	1	7.1	1.8
15	NAT2*5C/*7B	1	0.4	0	0	–	0	0	0	0	–
16	NAT2*6A/*7B	11	4.4	2	4.8	1.1	7	3.4	2	14.3	4.2
Total		80	32	20	47.6	1.5	69	34.2	9	64.3	1.9

*Abbreviations:* AT-DILI: antitubercular drug-induced liver injury; *NAT2*: *N*-acetyltransferase 2.

Strong LD was observed between rs1801280 and rs1799929 (r^2^= 0.89 [Southern Indian Site] and 0.80 [Western Indian Site], rs1801280 and rs1208 (r^2^= 0.90 [Southern Indian Site] and 0.81 [Western Indian Site] among the TB population of both the centres as shown in Figure 1. Good correlations were also observed between rs1041983 and rs1799930 (r^2^= 0.79 [Southern Indian Site] and 0.73 [Western Indian Site] and between rs1799929 and rs1208 (r^2^= 0.80 [Southern Indian Site] and 0.62 [Western Indian Site].

### 3.2. Review of literature on MAF of *NAT2* SNP of different TB populations

A relatively higher MAF (≥0.44) for rs1041983 was observed among TB patients of Southeast Asian countries such as Myanmar patients of Thailand [54], Vietnam [55] and Taiwan [23], as well as in the Southern part of India [56], whereas a lower MAF (≤0.03) was observed among TB patients of Latvia [57] and Ethiopia [58] as shown in Supplementary Figure 1A. The MAF for rs1801280 was relatively very high (≥0.35) among the TB patients of Brazil [59–62] and Ethiopia [58], whereas it was relatively very low (≤0.06) among TB patients from Southeast Asian countries such as Taiwan [23] and Vietnam [55] and low (0.18) among Russian TB patients [63] (Supplementary Figure 1B). The MAF reported for rs1801280 was 0.32 and 0.33 in Southern Indian [56] and Northern Indian [22] TB patients, respectively. Higher relative MAF (≥0.30) for rs1799929 was reported among several South American/Latin American TB patients such as Brazil [59,60,62], Peru [64], Venezuela [65] and Mexico [16,66] and lower MAF (≤0.06) among TB patients of Southeast and East Asian countries such as China [67], Mongolia [68], Taiwan [23] and Vietnam [55] and of Latvia [57] (Supplementary Figure 1C). The MAF of rs1799929 was reported to be 0.29 and 0.28 in Southern and Northern TB patients, respectively [22,56]. Higher MAF for rs1799930 (≥0.30) was observed in Southern [56], Northern [22] and Western Indian [69] TB patients. An MAF ≥0.26 was reported for TB patients of African countries such as Uganda [70] and Ethiopia [58], whereas South American/Latin American TB populations such as from Peru [64], Venezuela [65], Brazil [59,61,62] and Mexico [16,66] had a relatively lower MAF (≤0.17) for rs1799930 (Supplementary Figure 1D). South American/Latin American TB patients such as from Brazil [59–62], Venezuela [65] and Mexico [16,66] had relatively higher MAF (≥0.33) for rs1208, whereas a very relatively low MAF (≤0.06) was reported in TB patients from Southeast and East Asian countries such as Taiwan [23] and Vietnam [55], as well as from Latvia [57] (Supplementary Figure 1E). TB patients from Peru [64] and East and Southeast Asian countries such as Mongolia [68], Myanmar TB patients of Thailand [54], Taiwan [23] and Vietnam [55] were reported to have a relatively higher MAF (≥0.16) for rs1799931, whereas a relatively lower MAF (0.04) was reported for TB patients in Ethiopia [58] (Supplementary Figure 1F). The MAF of rs1799931 in the Southern Indian [56] and Northern Indian [22] TB patients was 0.07 and 0.11, respectively.

A relatively higher MAF for rs1041983 was observed among the AT-DILI group compared to those who did not develop AT-DILI in the TB patients of Brazil [61,62], Singapore [71], Indonesia [72,73] and China [74] as shown in Supplementary Figure 2A. TB patients with AT-DILI in Brazil [60–62] also had relatively higher MAF for rs1801280 compared to the non-AT-DILI group (Supplementary Figure 2B). TB patients who developed AT-DILI in Brazil [60,61], India [69], Tunisia [75], Mongolia [68] and Indonesia [72] had a higher MAF of rs1799929 (Supplementary Figure 2C). Higher MAF for rs1799930 was also observed among the AT-DILI group of TB patients from Southeast and East Asian countries [68,71–74,76], Brazil [61] and African countries compared to the non-AT-DILI group (controls) [58,75] (Supplementary Figure 2D). Two reports from TB patients in Brazil [60,62] also reported relatively higher MAF for rs1208 among the AT-DILI group (Supplementary Figure 2E). A relatively higher MAF for rs1799931 was also reported among the TB patients who developed AT-DILI from Singapore [71], Indonesia [72], China [74] as well as Brazil [62] (Supplementary Figure 2F) compared to the non-AT-DILI group.

Supplementary Table 1 describes the studies that have classified the *NAT2* acetylator into three categories, i.e. slow, intermediate and rapid by genotyping at least the six *NAT2* SNPs rs1041983, rs1801280, rs1799929, rs1799930, rs1208 and rs1799931 in the TB patients. Several studies in TB patients have shown that the *NAT2* slow acetylator varied from 22.7% to 58%, as shown in Supplementary Table 1.

### 3.3. ‘*All of Us*’ Research Program database

The MAF of the *NAT2* SNPs rs1041983, rs1801280, rs1799929, rs1799930 and rs1208 in the TB patients of the current study were in near ranges, whereas a slightly higher MAF was observed for rs1799931 in our study as compared to participants of South Asian genetic ancestry in the ‘All of Us’ Research Program database as shown in Figure 2. A relatively lower MAF for rs1801280, rs1799929 and rs1208 and a higher MAF for rs1799931 were observed for the East Asian population compared to other populations. The European and Middle Eastern participants had a near comparable and relatively higher MAF for rs1801280, rs1799929 and rs1208. The Latin American group had a relatively lower MAF for rs1799930 than the others. All the groups had a comparatively lower MAF of ≤0.16 for the rs1799931 compared to other *NAT2* SNPs.

## 4. Discussion

The current study explored the *NAT2* SNP diversity among TB populations of two different geographical regions of India and compared it to several other TB and AT-DILI populations and with a large-scale population of diverse genetic ancestries from the ‘All of Us’ Research Program. The MAF was near similar ranges for the six *NAT2* SNPs between the Southern and Western Indian TB patients. All six *NAT2* SNPs had a higher MAF among the TB patients who had AT-DILI as compared to the total TB population in the Southern Indian Site. However, a higher MAF only for the *NAT2* SNPs rs1041983, rs1799930 and rs1799931 were observed among the AT-DILI patients as compared to the total TB patients from the Western Indian Site. *NAT2* slow acetylators from the Western Indian Site had a relatively higher frequency of developing AT-DILI than the Southern Indian Site. We identified a strong LD between the *NAT2* SNPs rs1801280 and rs1799929 and between rs1801280 and rs1208 among both Southern and Western Indian TB patients.

South, Southeast and East Asian populations tend to have relatively higher MAF for rs1041983. Our study showed a relatively higher MAF of 0.43 and 0.41 for the rs1041983 from Southern and Western Indian TB patients which were in near comparable range to the previous report from South Indian TB patients [56] as well as South Asian participants from the ‘All of Us’ Research Program database. A relatively higher MAF (≥0.44) for rs1041983 was observed among the Southeast and East Asian TB patients [23,54,55]. The MAF for rs1041983 ranged from 0.22 to 0.39 for the South American/Latin American TB population (Supplementary Figure 1A). Two distinct clusters of participants, one in the MAF range of 0.32–0.33 (European, Latin American and Middle Eastern participants) and another in the MAF range of 0.42–0.45 (Our study results, South Asian, African and East Asian participants) were observed (Figure 2) for the rs1041983. A higher MAF for rs1041983 in the AT-DILI group was observed in both the Southern and Western Indian TB patients of our study relative to the MAF of total TB patients. rs1041983 was reported to affect the occurrence of AT-DILI significantly (OR: 2.39, 95%CI: 1.15–4.96, *p* = 0.019) among Indonesian paediatric TB patients [72]. Higher MAF among the AT-DILI group was reported among Southeast and East Asian [71–74] and South American TB populations [61,62] (Supplementary Figure 2A).

The MAF for the missense SNP rs1801280 for TB patients from the Southern and Western Indian Sites were in near comparable MAF range to the previous report from Southern and Northern Indian TB patients (Supplementary Figure 1B) [22,56] and South Asian and Latin American participants (Figure 2). The MAF for rs1801280 was relatively higher among Brazilian [59–62] and lower among Southeast and East Asian [23,55] and Russian [63] TB populations. A differential clustering of MAF for rs1801280 was observed, with a higher MAF observed for European and Middle Eastern participants, followed by a clustering of South Asian, current study and Latin American participants and a relatively lower and very low MAF observed for African and East Asian participants respectively. A higher MAF frequency for the AT-DILI group relative to the total TB population was observed in the Southern Indian TB patients in the current study, similar to reports from Brazilian TB patients [60,61]. The MAF for rs1799929 for the TB population of the Southern and Western Indian Sites were in near ranges to the previous reports among Southern [56] and Western Indian TB populations [69] respectively (Supplementary Figure 1C) and to the South Asian and Latin American participants (Figure 2). Higher MAF for rs1799929 was observed among South American/Latin American populations [16,59,60,62,64,66] and lower MAF among TB patients of Southeast and East Asian countries [23,54,55,67,68] and Latvia [57]. Similar to the rs1801280, a similar pattern of clustering of MAF of rs1799929 was observed, with a higher MAF observed for European and Middle Eastern participants, followed by a clustering of South Asian, current study, and Latin American participants and a relatively lower and very low MAF observed for African and East Asian population respectively. In our study, a relatively higher MAF for rs1799929 in the AT-DILI patients of the Southern Indian Site was observed, similar to a previous report from Indian TB patients [69]. Higher MAF for rs1799929 in the AT-DILI patients were also reported among South American [60,61], Southeast and East Asian [68,72] and African population [75] (Supplementary Figure 2C).

Indian and South American/Latin American populations tend to have relatively higher and lower MAF for rs1799930 respectively. The MAF for rs1799930 for TB patients from Southern and Western Sites were in near comparable ranges to the relatively high MAF reported from Southern Indian [56] and Northern Indian [22] TB patients (Supplementary Figure 1D) and to the South Asian participants (Figure 2). A relatively lower MAF for rs1799930 was observed among several South American/Latin American TB populations [16,59,61,62,64–66]. A relatively lower MAF was also observed among the Latin American participants of the ‘All of Us’ Research Program. A relatively higher MAF for rs1799930 among the patients who developed AT-DILI was observed in the AT-DILI group from Southern and Western Indian Sites, consistent with reports from Southeast and East Asian countries [68,71,74,76]. The MAF for rs1208 from TB patients of Southern and Western Sites were in near comparable ranges with few reports of South American/Latin American TB patients (Supplementary Figure 1E) and South Asian and Latin American participants (Figure 2). A relatively higher MAF for rs1208 in South American/Latin American populations (16, 59–62, 66) and lower MAF among Southeast and East Asian [23,55] and Latvia [57] TB patients was observed. As with rs1801280 and 1799929, a relatively very low frequency for rs1208 was observed for the East Asian participants and a relatively higher MAF for Middle Eastern and European participants of the ‘All of Us’ Research Program. A higher MAF was reported for rs1208 in the AT-DILI group TB patients from the Southern Indian Site in our study, as observed in reports from the Brazilian TB population [60,62] (Supplementary Figure 2E) in our study. Compared to the other *NAT2* SNPs, a relatively lower MAF was observed for rs1799931 in our Study Sites and other TB populations and participants of the ‘All of Us’ Research Program. A relatively higher MAF was observed among the Southeast and East Asian TB population [23,54,55,68] and Peruvian [64] and lower in the Ethiopian population [58] for rs1799931. The East Asian participants had a relatively higher MAF for rs1799931 in the ‘All of Us’ Research Program. A higher MAF was observed for rs1799931 in the AT-DILI patients from both the South and Western Indian Sites, similar to other reports from South American, Southeast and East Asian TB populations [62,71,74].

A minor allele having a frequency of ≥0.05 (5%) and <0.01 (1%) is termed a common variant and a rare variant respectively [77,78]. All six *NAT2* SNPs were identified to be common variants among the TB patients of both Southern and Western Indian Sites, as well as in the literature data on the Indian TB population, suggesting the need for a six SNP *NAT2* genotyping panel in the Indian TB population. Our study provides strong evidence that geographically close countries and populations with similar genetic ancestry generally shared common MAF characteristics for the six *NAT2* SNPs. Similar MAF characteristics were also observed for TB patients from the literature reviews and participants who had similar genetic ancestry from the ‘All of Us’ Research Program database. For instance, Southeast East and East Asian TB populations and participants of the East Asian genetic ancestry generally tend to have relatively higher MAF for rs1041983 and rs1799931 and a very low MAF for rs1801280, rs1799929 and rs1208. Though SNPs with MAF ≥0.05 are preferentially used for most of the case-control studies of human diseases, rare functional SNPs have been potentially implicated in several disease causation [79]. Hence future studies focussing on the trade-off between cost and benefits should be assessed in larger sample-sized populations where these SNPs occur in low frequency to decide on their exclusion from the genotyping panels in settings having cost constraints. Literature review and database comparison showed that the Indian TB population from Southern and Western India shared near common MAF characteristics with several South American/Latin American TB populations and Latin American genetic ancestry respectively concerning the *NAT2* SNPs rs1801280, rs1799929, rs1208 and rs1799931. Also, TB patients from Southeast/East Asian Countries and participants of East Asian ancestry have a higher MAF for rs1041983 and rs1799931 compared to TB patients from Southern and Western Indian Sites. Inter and intra-country discrepancies have been observed in the distribution of MAF of the six *NAT2* among a few AT-DILI and non-DILI TB populations, warranting a region/population-specific large sample size approach for interrogating *NAT2* SNP as potential biomarkers for AT-DILI. All the detected SNPs except for rs1799931 were found in HWE, which was similarly reported in an Indian study on patients with TB and normal healthy volunteers [80]. Several studies have shown that there exists an LD between rs1801280 and rs1799929 [64,80–83], rs1801280 and rs1208 [80,82,83], rs1799929 and rs1208 [80, 82, 83] and rs1041983 and rs1799930 [80]. This could probably explain the trend of relatively higher MAF for European and Middle Eastern participants and lower MAF for East Asian participants for rs1801280, rs1799929 and rs1208 in the ‘All of Us’ Research Program.

About one-third of the TB populations of both the study centres were *NAT2* slow acetylator phenotype, and *NAT2* slow acetylator had a higher frequency of developing AT-DILI in our study population. Several other *NAT2* genotyping studies using the minimum of six SNP panels assessed in the current study reported the percentage of *NAT2* slow acetylators as 31.2% in the Vietnamese TB population [55], 38.3% in the Northern Indian TB population [22], 27.2% and 31.9% in Brazilian TB population [61,62]. Reports from other population studies have shown the proportion of *NAT2* slow acetylators to be 39.6% among Mexican healthy volunteers [16] and among Central Asian populations such as 26% in Kirghizs, 32% in Uzbeks and 35% in Kazakhs [84]. Higher frequencies of *NAT2* slow acetylators among TB patients who developed AT-DILI have been reported in other populations [61]. *NAT2* slow acetylators are at higher risk for developing several antitubercular drug-induced ADRs [85,86]. TB patients with *NAT2* slow acetylator status have been reported to display a trend of association with a mild increase in liver enzymes [87]. TB patients with the *NAT2* SNPs rs1801280, rs1799929 and rs1208 had higher liver function test (LFT) values such as ALP, ALT, AST, total and direct bilirubin and higher odds of developing AT-DILI [88]. *NAT2* genotyping may also reduce TB patients’ risk of early treatment failure [35]. The *NAT2* SNPs and genotype implicated as risk factors in a particular TB population may be utilized as a preemptive biomarker for stratifying the patients for more frequent serial monitoring of the LFT, particularly during the first two months of ATT, to reduce the incidence of AT-DILI.

Our results reveal that the diversity of the *NAT2* gene concerning SNPs, alleles and genotypes could occur within a country’s population and across countries within a continent. Considerable diversity was also observed in MAF of the *NAT2* SNPs in the TB patients experiencing AT-DILI in the TB patients of our two geographically diverse Study Sites as well as among different TB populations from the literature review. This *NAT2* genetic diversity across geographical regions may be attributed to the shift from a hunter-gatherer lifestyle to the adaptation of agricultural practices, gene-environment interactions and gene flow [84,89,90]. The *NAT2* phenotypes derived from these combinations of these *NAT2* SNPs could significantly influence the isoniazid pharmacokinetics and the development of AT-DILI [91]. The higher MAF of the *NAT2* SNPs and the potential association of the SNPs and phenotype with AT-DILI reinforces the need for consideration of *NAT2* pharmacogenomics as a potential preemptive biomarker for monitoring LFT and AT-DILI in TB treatment. Most of the research investigations including ours suggest that more than a quarter of the TB population are *NAT2* slow acetylators, warranting the need for introspection into the incorporation of *NAT2* genotyping as a potential diagnostic tool in the clinical management of TB. By identifying patients with the *NAT2* slow acetylator genotype, healthcare providers can predict an increased risk of AT-DILI. This allows for personalized TB treatment strategies, such as adjusting drug dosages or developing a closer LFT monitoring strategy to minimize adverse effects and optimize efficacy. The lack of ethnic-specific information and sampling from other geographical areas such as Northern, Eastern and Central parts of India is another limitation in providing a pan-Indian perspective on *NAT2* heterogeneity in TB and AT-DILI patients. Other study shortcomings include that not all studies reported/genotyped all six *NAT2* SNPs and categorized the *NAT2* acetylator into three phenotypes. The majority of the literature on *NAT2* SNP frequency in TB patients was reported from Southeast and East Asian, Indian and South American countries. There is a paucity of literature on *NAT2* genotyping from several other high-burden TB countries, which limited us to presenting a true global picture of *NAT2* heterogeneity. Nevertheless, the vast amount of data collected enabled us to provide a comprehensive picture of the *NAT2* genomic landscape across different parts of the world, thus building up a useful resource of frequency data for further studies focusing on *NAT2* molecular epidemiology and developing *NAT2* genotype as a potential biomarker for LFT monitoring during isoniazid-based ATT.

Further well-designed multicentric studies with *NAT2* genotyping among large and geographically and ethnically diverse TB and AT-DILI populations with a dynamic follow-up could overcome the limitations of our research and several other literatures on TB patients that were included in the current study of having smaller AT-DILI populations. Randomized controlled trials assessing the benefits of *NAT2* pharmacogenomic guided LFT monitoring and isoniazid dosing regimen are imperative for deriving conclusive evidence on the benefits of incorporating *NAT2* genotyping in the clinical management of TB patients.

## 5. Conclusion

The current study provides an overview of the pharmacogenomic landscape of *NAT2* SNPs among TB and AT-DILI patients across two different geographical regions of India, comparing it to TB populations from other parts of the world and populations with different genetic ancestries from the ‘All of Us’ Research Program database. Several geographically close regions and populations with similar genetic ancestries shared near similar *NAT2* SNP MAF characteristics, though diversities were also observed within a country population. All the six *NAT2* SNPs rs1041983, rs1801280, rs1799929, rs1799930, rs1208 and rs1799931 were identified as common variants in most of the population. Though ample evidence is available for using *NAT2* phenotype as a potential biomarker for AT-DILI, further validation of the different *NAT2* SNPs as risk factors for AT-DILI are required due to the significant MAF diversity observed among AT-DILI and controls within a region and across various regions. *NAT2* preemptive pharmacogenomic guided therapy could be a potential tool for monitoring and risk stratification of ADRs such as AT-DILI in TB patients, exemplified by the relatively higher proportion of AT-DILI among the *NAT2* slow acetylators in our study.

## Supplementary Material

Supplemental Material

## Data Availability

The data that support the findings of this study are available from the corresponding author upon reasonable request.
