# Supplementary material for: Pharmacogenomic heterogeneity of N-acetyltransferase 2: a comprehensive analysis of real world data in Indian tuberculosis patients and from literature and database review
Source: Ann Med. 2025 Mar 26;57(1):2478316. doi: 10.1080/07853890.2025.2478316 (PMC11948353; doi:10.1080/07853890.2025.2478316)
Supplement: Supplemental Material [file IANN_A_2478316_SM3012.zip › Suppl/Supplementary_Figure caption.docx]

***Supplementary Figure 1.*** MAF of NAT2 SNPs among different TB populations.

Abbreviations: MAF= Minor allelic frequency, NAT2= N-acetyltransferase 2, SNP= Single nucleotide polymorphism.

Footnote: The top 25% of the TB population with high MAF have been highlighted with green boxes and the bottom 25% have been highlighted with pink boxes.

***Supplementary Figure 2.*** MAF of different NAT2 SNPs among AT-DILI (cases) and non-ATDILI (controls) among different TB pop.

Abbreviations: AT-DILI= antitubercular drug-induced liver injury, MAF= Minor allele frequency, NAT2= N-acetyltransferase 2, SNP= Single nucleotide polymorphism

Footnote: The TB population where AT-DILI (cases) have a higher MAF difference of ≥ 0.05 over the non-AT-DILI (controls) have been highlighted with green boxes.
