# Supplementary material for: Pharmacogenomic heterogeneity of N-acetyltransferase 2: a comprehensive analysis of real world data in Indian tuberculosis patients and from literature and database review
Source: Ann Med. 2025 Mar 26;57(1):2478316. doi: 10.1080/07853890.2025.2478316 (PMC11948353; doi:10.1080/07853890.2025.2478316)
Supplement: Supplemental Material [file IANN_A_2478316_SM3012.zip › Suppl/Supplementary_Table_1 (1).docx]

**Supplementary Table 1.** *NAT2* phenotype distribution across different TB populations

| Sl. No | Author, year [reference] | Sample size | Country of study | *NAT2* phenotype %  [SA/IA/RA] |
| --- | --- | --- | --- | --- |
|  |  |  |  |  |
| 1 | Santos et al, 2013 [62] | 270 | Brazil | 31.9/48.5/19.6 |
| 2 | Possuelo et al, 2008 [61] | 254 | Brazil | 27.2/39/33.9 |
| 3 | Heinrich et al, 2016 [59] | 115 | Brazil | 54.6/34.3/11.1 |
| 4 | El-Jaick et al, 2022 [60] | 162 | Brazil | 57.3/34.0/8.7 |
| 5 | Salazar-González et al, 2014 [16] | 40 | Mexico | 32.5/32.5/35 |
| 6 | Verhagen et al, 2014 [65] | 30 | Venezuela | 40/43.3/16.7 |
| 7 | Levano et al, 2021 [64] | 395 | Peru | 46.8/38.2/14.9 |
| 8 | Hemanth et al, 2017 [56] | 326 | India | 58/35/7 |
| 9 | Ho et al, 2013 [23] | 348 | Taiwan | 22.7/52.3/25 |
| 10 | Le et al, 2019 [55] | 136 | Vietnam | 31.6/42.7/25.7 |
| 11 | Krasnova et al, 2020 [63] | 132 | Russia | 22.7/58.3/18.9 |
| 12 | Igumnova et al, 2016 [57] | 85 | Latvian | 51.8/43.5/4.7 |

Abbreviations: IA= Intermediate acetylator, *NAT2*= *N*-acetyltransferase 2, RA= Rapid acetylator, SA= Slow acetylator
