# Supplementary figures and images for: Pharmacogenomic heterogeneity of N-acetyltransferase 2: a comprehensive analysis of real world data in Indian tuberculosis patients and from literature and database review
Source: Ann Med. 2025 Mar 26;57(1):2478316. doi: 10.1080/07853890.2025.2478316 (PMC11948353; doi:10.1080/07853890.2025.2478316)

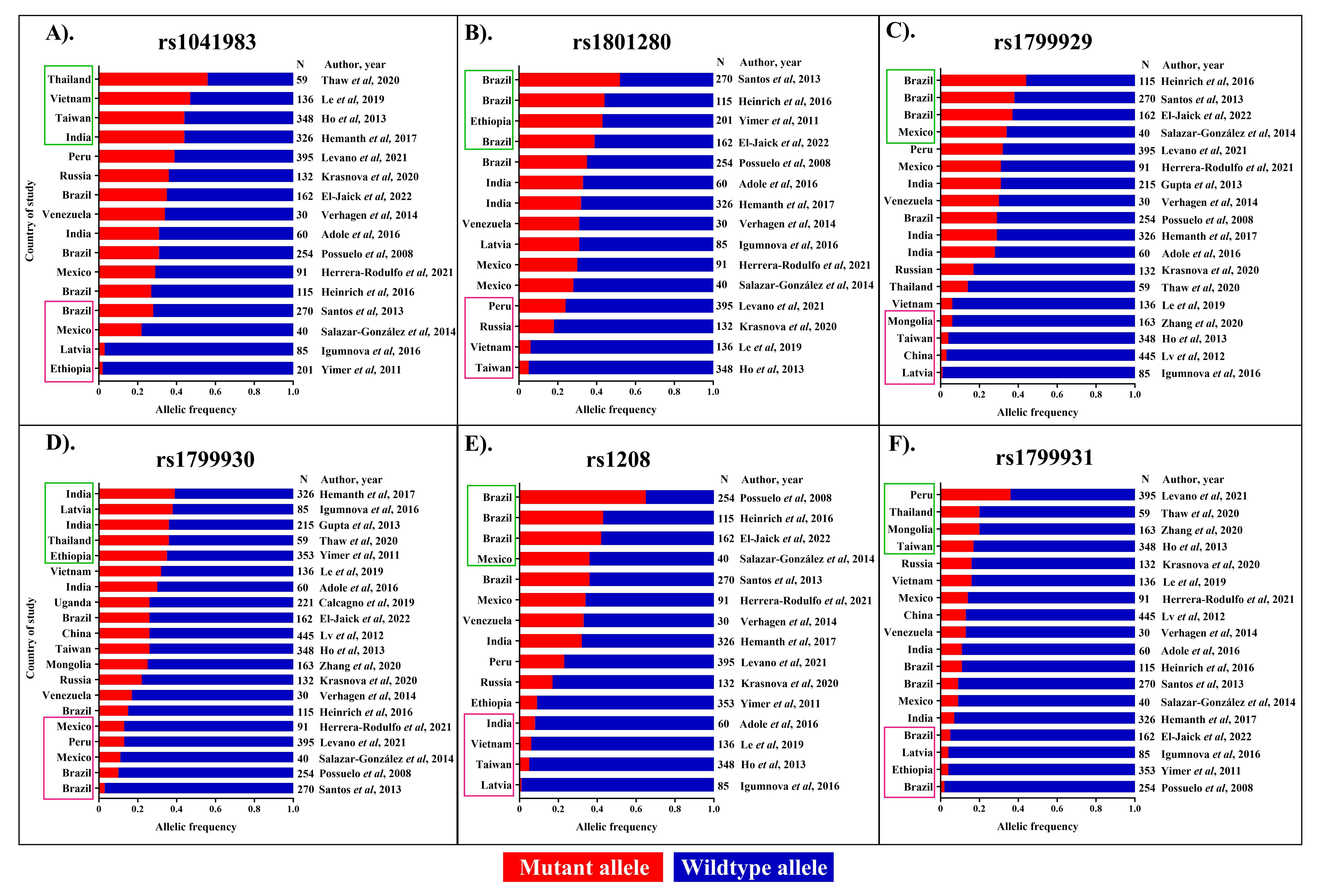

Supplement: Supplemental Material [file IANN_A_2478316_SM3012.zip › Suppl/Supplementary_Figure_1.jpeg]

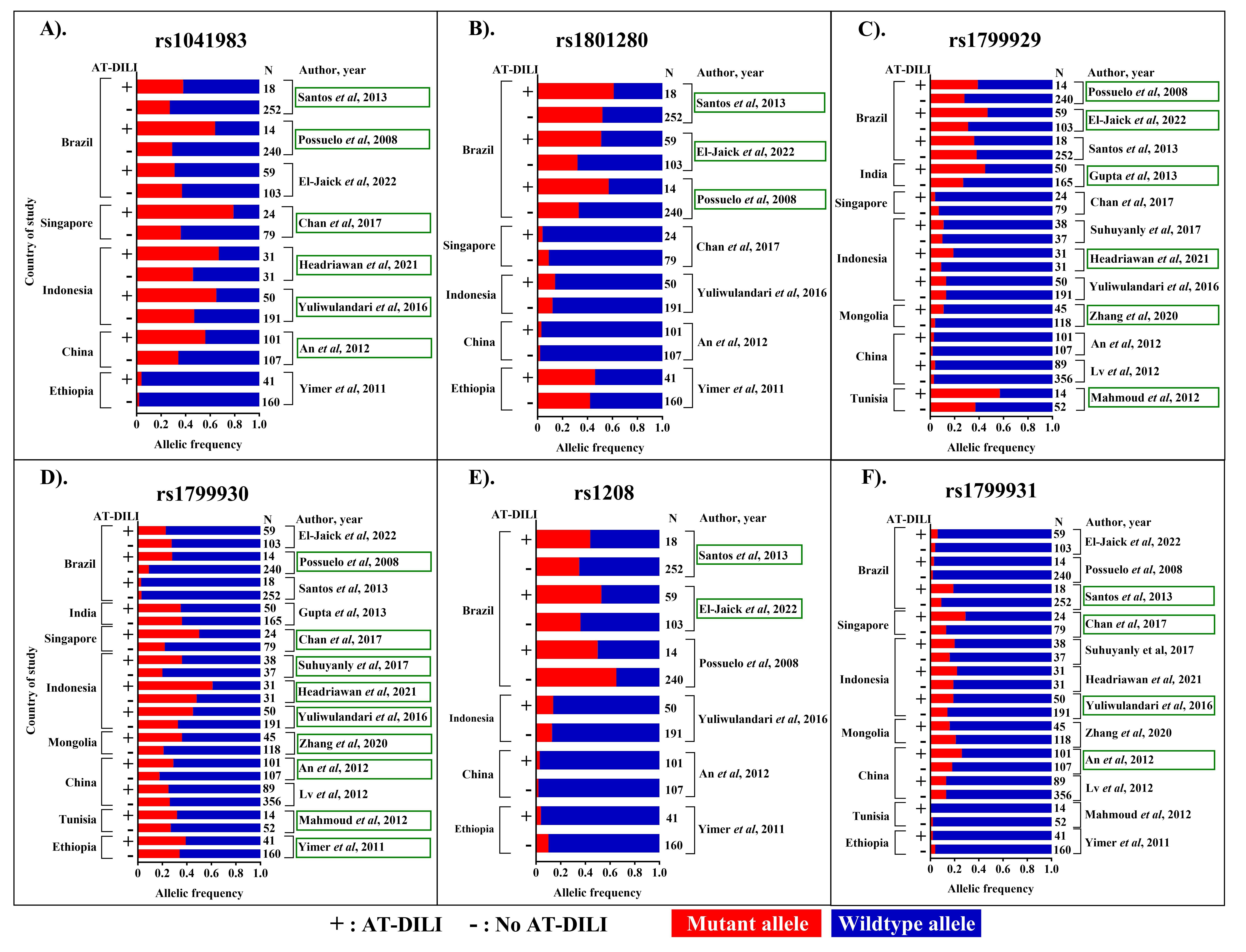

Supplement: Supplemental Material [file IANN_A_2478316_SM3012.zip › Suppl/Supplementary_Figure_2.jpeg]
